# IL-33/IL-31 Axis in Osteoporosis

**DOI:** 10.3390/ijms21041239

**Published:** 2020-02-13

**Authors:** Massimo De Martinis, Maria Maddalena Sirufo, Mariano Suppa, Lia Ginaldi

**Affiliations:** 1 Department of Life, Health and Environmental Sciences, University of L’Aquila, 67100 L’Aquila, Italy; maddalena.sirufo@gmail.com (M.M.S.); lia.ginaldi@cc.univaq.it (L.G.); 2 Allergy and Clinical Immunology Unit, Center for the Diagnosis and Treatment of Osteoporosis, AUSL 04 64100 Teramo, Italy; 3 Department of Dermatology, Hôpital Erasme, Université Libre de Bruxelles, 1070 Brussels, Belgium; mariano.suppa@erasme.ulb.ac.be

**Keywords:** osteoporosis, IL-31, IL-33, bone, osteoimmunology, immunity, allergy

## Abstract

The study of the immunoskeletal interface has led to the discovery of numerous cytokines involved in the regulation of bone remodeling, providing valuable information on the pathogenesis of osteoporosis. The role of inflammatory cytokines of the Th1 and Th17 profile in osteoporosis is well known. Here we focus on two newly discovered Th2 cytokines, IL-31 and IL-33, whose implications in osteoporosis are recently emerging. Clinical and experimental observations suggest an important role of the IL-33/IL-31 axis in osteoporosis. IL-33 induces IL-31 secretion by Th2 cells and inhibits RANKL-dependent osteoclastogenesis, thus counteracting bone loss. IL-31 influences Th1/Th17 osteoclastogenetic inflammation and limits Th2 osteoprotective processes, thus favoring osteoporosis. Better knowledge of the role of IL-31 and IL-33 and their receptor complexes in osteoporosis could provide an interesting perspective for the development of new and more effective therapies, possibly with less side effects.

## 1. Osteoporosis and the Cytokine Regulation of Bone Remodeling

Osteoporosis is a predominantly female pathology of the skeleton characterized by loss of bone mass, decreased bone mineral density (BMD), and derangement of bone microarchitecture, which lead to a compromised physical strength of the bone and increased fragility of the skeleton, exposing patients to a greater risk of fractures on minor trauma [1]. Osteoporosis prevalence worldwide is high, affecting more than 200 million people, and the fractures it causes represent a major determinant of morbidity, disability, and mortality in older people [2]. Osteoporosis is a multi-factorial disease whose etiopathogenetic mechanisms variously overlap. Primary (post-menopausal and senile) and secondary osteoporosis (caused by various drugs and pathologies) can be distinguished [3,4,5]. Bone is a metabolically active tissue composed of a mineralized protein matrix and specialized cells (osteoblasts, osteocytes, and osteoclasts) [6]. The skeleton is continuously remodeled throughout life by a process of bone resorption, mediated by osteoclasts (giant cells of the myeloid lineage, containing lysosomal enzymes), followed by bone formation, mediated by osteoblasts (osteocyte precursors derived from mesenchymal stem cells) [7]. When excessive bone resorption occurs and/or in the presence of inadequate bone formation, an osteoporosis condition develops with an increased risk of fractures [8]. The various phases of bone formation and resorption coexist in a dynamic equilibrium, finely regulated by a complex cytokine network [9,10]. The mutual influence between the immune system and bone (the immunoskeletal interface) impacts on bone turnover both in physiological and pathological conditions through intense crosstalk between immune and bone cells [11]. This dialogue, although still partially undeciphered, represents a promising research field, since its understanding could provide new insights for the design of targeted therapeutic strategies for osteoporosis [12]. Most of the pathologies causing osteoporosis are characterized by a chronic inflammatory background [13,14,15]. The menopausal estrogen decline and the aging process induce osteoporosis, mainly increasing the production of inflammatory cytokines that exert osteoclastogenic properties [9]. Local and systemic bone loss also represent the hallmark of inflammatory rheumatic conditions and reflect the close interaction between bone and immune system, leading to osteoclast hyperactivation with consequent uncoupling of bone formation and resorption [16,17,18]. The central signal pathway in bone resorption is the system of the receptor activator of NF-kB ligand (RANKL), mainly expressed by osteoblasts, that binds to its receptor RANK on the osteoclast precursors and mature osteoclasts, thus inducing osteoclastogenesis and bone resorption [19,20]. However, RANKL is expressed not only by osteoblasts but also by a variety of other cell types, including chondrocytes and osteocytes, which are embedded in matrix. There is evidence that RANKL derived from osteocytes is responsible for the bone loss associated with unloading, whereas RANKL produced by osteoblasts or their progenitors does not contribute to adult bone remodeling. Matrix resorption is therefore controlled by cells embedded within the matrix itself [21,22]. Each cytokine of the complex network of regulatory factors involved in bone remodeling has pleiotropic functions and exerts different effects depending on the target cells and the influence of other cytokines in the specific microenvironment [23,24]. Osteoclastogenic cytokines, such as interleukin (IL)-6, IL-17, interferon (IFN)-γ and tumor necrosis factor (TNF)-α, the macrophage-colony stimulating factor (M-CSF) and monocyte chemoattractant protein-1 (MCP-1), promote bone resorption and inhibit osteoblasts, whereas other cytokines, such as IL-4, IL-10, transforming growth factor (TGF)-β, and IL-12, suppress osteoclasts and promote osteogenesis. It is commonly believed that most of the cytokines of the T helper 1 (Th1) profile are osteoclastogenic, whereas Th2 cytokines exert osteoanabolic and protective functions on the bone [11,25]. For example, the therapy with anti-TNF-α monoclonal antibodies in rheumatoid arthritis blocks periarticular bone erosions. IL-6 is an essential osteoclastogenic factor produced by neoplastic plasma cells in multiple myeloma and is also a major predictor of bone loss in menopausal osteoporosis [26]. On the other hand, Th2 cells are commonly believed to be responsible for anti-inflammatory activity in various Th1 mediated diseases, and therefore, at least in some conditions, could counteract the osteoclastogenic functions of other inflammatory cells.

Osteoporosis could be therefore considered an age-related inflammatory disease mediated by Th1/Th17 cells. Pro-inflammatory cytokines induce both low-grade inflammation and impaired DNA repair, leading to cell senescence, biological aging, and the peculiar immune remodeling characterizing immunosenescence. The hyperproduction of pro-inflammatory cytokines belonging to the Th1 profile, such as TNF-α, IL-1, IL-6, IL-17, constitutes the background of both secondary and primary osteoporosis. In contrast, cytokines of the Th2 profile, such as IL-4, and regulatory cytokines, such as IL-10, have a predominantly osteoanabolic function and are usually considered protective against osteoporosis [2,9,26]. Furthermore, mice without T lymphocytes are protected from the catabolic activity of PTH, and Th2 lymphocytes lower the RANKL/OPG ratio by inhibiting bone loss. Low concentrations of Th2 cytokines IL-4 and IL-10 are present in synovial fluid and peripheral blood of patients with osteoarthritis. IL-4 inhibits bone resorption both in vivo and in vitro, thus clearly suggesting an osteoprotective role of the Th2 lymphocyte profile in osteoporosis [27]. On the other hand, T regulatory lymphocytes (Tregs), which are among the main inhibitors of bone resorption, induce the production of the TGF-β1, IL-4 and IL-10 cytokines. Treg cells also inhibit Th17 lymphocytes and Th17 mediated inflammatory bone loss. In addition, Tregs suppress monocyte differentiation into osteoclasts, both in vitro and in vivo [28].

Cytokines have been therefore classified according to their stimulatory or inhibitory effect on proliferation and differentiation of osteoclasts. Inflammatory cytokines, mostly of the Th1/Th17 profile, exert stimulating effects on osteoclastogenesis [29], whereas Th2 cytokines have been reported to have an inhibitory effect on the proliferation and differentiation of osteoclasts [30]. However, there are some cytokines that exhibit variable and pleiotropic effects on bone turnover, depending on multiple factors. For example, the Th1 cytokine IFN-γ plays conflicting roles in osteoclastogenesis, the final effect depending on the balance of direct or indirect effect, as well as the stage of osteoclast differentiation. IFN-γ inhibits the early differentiation of osteoclasts by targeting the RANK–RANKL pathway, whereas it promotes the fusion of mononucleated osteoclasts in the late stage of osteoclast formation. Moreover, it indirectly increases osteoclastic factors by activating immune responses [31]. Therefore, the pleiotropic cytokine IFN-γ functions as an anti-osteoclastogenic cytokine in physiological conditions of bone turnover, by binding to its specific receptor on osteoclasts and inducing TRAF6 proteasomal degradation with consequent inhibition of the transduction signal mediated by RANKL. However, in postmenopausal osteoporosis, inflammation or infections, the net effect of IFN-γ, is biased towards bone resorption via antigen-driven T cell activation and RANKL production [9].

Also, the regulation of macrophage polarization is of paramount importance in enhancing bone formation and maintaining bone homeostasis [32,33]. In particular, M1 macrophages, through immunomodulatory factors (cytokines and chemokines) secretion, stimulate pro-inflammatory cytokine expression and bone loss, whereas M2 macrophages, through vascular endothelial growth factor (VEGF) and bone morphogenetic protein (BMP)-2 secretion, exert anti-inflammatory functions promoting bone repair. Taken together, these findings suggest that osteoporosis is determined by a complex overlap of different factors.

Recently evidence has emerged of the role of two new cytokines of the Th2 profile, IL-31 and IL-33, in bone remodeling and osteoporosis [34,35,36]. Since the discovery of IL-31 and IL-33, their possible relationship in the context of osteoimmunology has emerged. The IL-33/IL-31 axis had already been highlighted as a potential inflammatory pathway in chronic inflammatory diseases. The expression of one molecule is capable of inducing the production of the other, thus generating an amplification circuit of the phlogistic process with consequent disease development [37]. However, although the role of the IL-33/ST2 axis in Th2/IL-31 and Th17 immune responses, characterizing the development of allergic respiratory diseases, has been recently clarified [38,39], the relationship between IL-31 and IL-33 in osteoporosis is quite peculiar and the data from the literature are often contradictory.

## 2. The Role of IL-31 in the Immunoskeletal Interface

The Th2 pleiotropic cytokine IL-31 exerts an important role in immunity and inflammation. It is a helical molecule belonging to the gp130/IL-6 family of cytokines, which also includes IL-6 and oncostatin M. IL-31 gene expression is regulated by both IL-4 and IL-33 and its secretion from mast cells is augmented by immunoglobulin E and neuropeptide substance P stimulation [40,41]. The receptor of IL-31 (IL-31R) is a heterodimeric receptor, consisting of two subunits, oncostatin-M receptor β (OSMR) and IL-31 receptor α (IL-31RA), expressed on different cell types, including IL-31 activated monocytes and myeloid progenitor cells. IL-31RA interacts with SH-2 (Src homology 2), whereas OSMR binds the adaptor protein Shc through phosphorylated tyrosines of the OSMR intracellular domain [29].

IL-31, mainly produced by activated CD45RO+ T lymphocytes skewed toward a Th2 phenotype, acts on different tissues, performing multiple regulatory functions on cell proliferation and tissue remodeling, and also induces the production of inflammatory cytokines. Moreover, IL-31R activation upregulates the number of immature subpopulations of hemopoietic cells and is involved in the positive regulation of their cycling status [41,42].

IL-31 exerts central roles in the pathogenesis and progression of atopic dermatitis and chronic spontaneous urticaria. IL-31, released by CD45RO+ Th2 lymphocytes but also by macrophages, dendritic and eosinophilic cells, mediates the itching in patients suffering from allergic diseases, whereas IL-33, secreted by inflamed or damaged tissue, functions as a local early warning signal [43]. However, although IL-31 primarily induces Th2 responses and allergic manifestations [44], it also suppresses Th2 inflammation [45].

**Figure 1:**
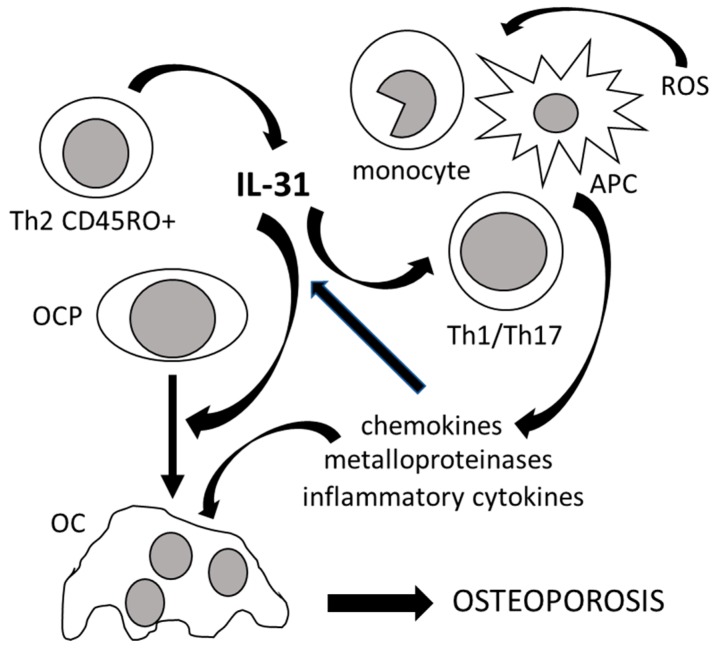
The role of IL-31 in bone remodeling. IL-31, mainly produced by T helper 2 memory cells (Th2 CD45RO+), promotes osteoclastogenesis by inducing the differentiation of osteoclast progenitors (OCP) into mature osteoclasts (OC). IL-31, in synergy with reactive oxygen species (ROS), also stimulates osteoclastogenesis indirectly by inducing antigen-presenting cells (APC), monocytes, and T helper 1 and 17 lymphocytes (Th1/Th17) to produce chemokines, metalloproteinases, and inflammatory cytokines, which in turn increase the production of IL-31. All of these events lead to the development of osteoporosis.

The IL-31 signal system is involved in maintaining homeostasis of myeloid progenitors, which are also the precursors of bone resorbing osteoclasts [46]. IL-31 induces the activation of monocytes and macrophages and increases the transcription of proinflammatory cytokine genes leading to the production of osteoclastogenic cytokines, in addition to matrix metalloproteinases and chemokines [35,47]. Finally, it also regulates Th1/Th17 lymphocyte differentiation and antigen-presenting cell function [48]. Figure 1 illustrates the role of IL-31 in bone remodeling.

Many of the transcription factors and cytokines pathogenetically involved in the development of osteoporosis are regulated by IL-31. For example, signaling pathways involved in both bone remodeling and inflammation, such as Janus-activated kinase (JAK) and molecules downstream of this signaling pathway, including important signal transducers and activators of transcription (STAT), as well as NF-kB, PI3K/AKT (phosphatidylinositol 3′-kinase/protein kinase), and MAPK (mitogen activated protein kinase) pathways, are regulated by IL-31. STAT-1 and STAT-3 mediate intracellular events leading to proliferation and activation of osteoclasts [49]. The activation of the receptor complex by IL-31 also signals via additional transcription factors involved in bone turnover regulation, including extracellular signaling regulated kinases 1 and 2 (ERK1/ERK2) [50,51]. In vitro studies showed that IL-31 enhances the production of chemokines, metalloproteinases, and pro-inflammatory cytokines essential for the recruitment, differentiation, and function of osteoclasts [52,53,54]. The IL-31/IL-31RA interaction, through the mediation of STAT1/3/5 and MAPK activation, starts signals leading to pro-inflammatory cytokine and chemokine expression, that exert central roles in both Th2 cytokine-mediated inflammatory diseases and bone resorption, leading to osteoporosis. STAT1 activation induces CCL-17 and CCL-22 chemokines, which guide the localization of osteoclasts and their precursors in bone resorption sites, where, upon the activation by pro-inflammatory cytokines and RANKL, they complete their maturation [55]. The IL-31-induced ERK1/ERK2 signaling influences cartilage homeostasis and osteochondroma formation [56]. IL-31 can also significantly upregulate the gene expression and protein levels of epidermal growth factor (EGF) and VEGF, both involved in angiogenesis, tumor growth, and metastasization. Therefore, according to the relationship between inflammation and angiogenesis, it has been hypothesized a role of IL-31 in tumorigenesis and bone metastasis processes [57]. IL-31 control of cell proliferation via the STAT receptor pathway, in turn activated by other IL-6 family cytokines, likely exerts potential roles in bone tissue damage and repair as well as in tumorigenesis and bone metastases. A malfunction of these IL-31 shared signaling pathways is involved in several diseases in addition to osteoporosis, including diabetes, cancer, neurological and cardiovascular diseases, all in some way also related to impaired bone remodeling and osteoporosis.

The important role of IL-31 in bone metabolism is also suggested by the bone phenotypes of IL-31/IL-31R knockout mice, which are characterized by increased production of Th2 cytokines, indicating a regulatory role for IL-31–IL-31R interaction in limiting Th2 inflammation in favor of a Th1/Th17 profile [29]. The lack of IL-31 signaling causes the failure to activate multiple JAK-STAT and MAPK signaling pathways and, in particular STAT3, which is central in RANKL-mediated osteoclastogenesis [58]. IL-31RA-deficient mice also exhibit elevated responsiveness to oncostatin M, whose overexpression induces a phenotype of osteopetrosis [46].

In summary, IL-31 appears to be a key mediator among the cytokines involved in bone remodeling. By acting on various cell types, IL-31 strongly induces the release of IL-1β, IL-6, and the chemokines CXCL1, CXCL8, CCL2, and CCL18 [59]. IL-31 could, therefore, function indirectly through the induction of proinflammatory cytokines involved in the recruitment of osteoclast precursors and immune-mediated bone resorption. Some functions of IL-31 are similar to those of IL-17 and both have reciprocal additive effects in inducing inflammatory cytokines and increasing bone resorption [60]. On the other hand, inflammatory cytokines, especially IFN-γ, IL-1β, and TNF-α, can, in turn, increase the expression of IL-31 and its receptors. During aging, in addition to proinflammatory cytokines, the production of reactive oxygen species (ROS) is also increased. The expression of IL-31 in monocytes, dendritic cells, and T lymphocytes can be induced by ROS, enhancing the age-related bone resorption [61].

## 3. The Role of IL-33 in the Immunoskeletal Interface

IL-33 is a Th2 cytokine mainly expressed by stromal cells following proinflammatory stimulation. It functions as a traditional cytokine, as an alarmin, and as a nuclear factor able to control gene transcription [34]. As alarmin, IL-33 is released upon cell injury and after cell death, participating in stress responses and resulting in the induction of other cytokines [62]. IL-33 also acts extracellularly as a ligand for the IL-1 receptor family member ST2 (IL-1R-like 1), and the binding of IL-33 to ST2 further activates downstream pathways, leading to increased transcription of Th2 cytokines, including IL-31 [63]. ST2 is mainly expressed by cells of innate immunity, mast cells, and Th2 lymphocytes, although recently it has also been found on natural killer (NK) cells, Treg and Th1 lymphocytes [64]. Different variants of the ST2 junction lead to the production of different receptor forms, including a soluble form (sST2) that acts as a decoy receptor by binding the extracellular IL-33, thus preventing its interaction with the membrane receptor. Accordingly, recombinant sST2 antagonizes the pro-inflammatory effect of IL-33 in respiratory allergy [36,37,38].

IL-33 downstream signaling requires the accessory receptor IL-1R3 to function. The ST2/IL-1R3 transduction signal leads to the activation of NF-κB, c-Jun N-terminal kinase (JNK), and MAPK [44,45]. IL-33 activates naive T cells and promotes their maturation towards a Th2 phenotype, leading to the release of Th2 type cytokines and chemokines. IL-33 influences various adaptive and innate immune cells, including eosinophils, mast cells, type 2 innate lymphoid cells (ILC2), and Th2 lymphocytes [42,43,44]. Via the NF-kB and MAPK signaling pathways, IL-33 induces the production of IL-5, IL-13 and CCL5, CCL17, CCL24 chemokines [57,65]. The response to IL-33 depends on the cell type, the cellular composition of the tissue microenvironment, and the presence of co-stimulating cytokines [66,67,68,69].

Upon cell damage and loss of cellular integrity, IL-33 is released from the nucleus, acting as a dual-function alarmin, similarly to high-mobility group box 1 (HMGB1) and IL-1 α [69]. However, unlike other alarmins, IL-33 is rapidly oxidized in the extracellular space. The oxidation of IL-33 represents a crucial mechanism of downregulation of its function [70]. Although previously linked to the initiation and chronicization of inflammatory bone resorption, recently, a role in resolving inflammation and bone loss has been suggested [30]. 

**Figure 2:**
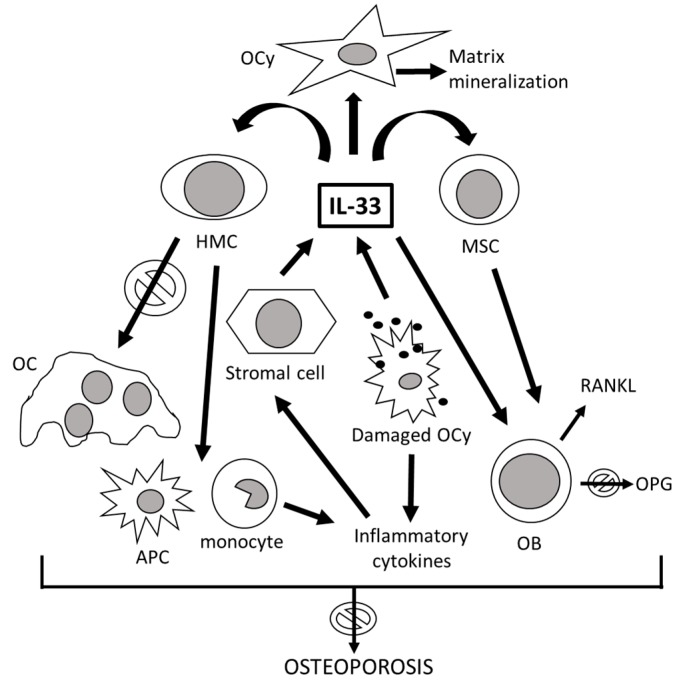
The role of IL-33 in bone remodeling. After inflammatory stimulation, the stromal cells produce IL-33, which directly blocks osteoclast (OC) formation from hemopoietic myeloid cells (HMC), shifting the osteoclast precursor differentiation towards cytokine-producing macrophages and antigen-presenting cells (APC). Damaged osteocytes (OCy), in addition to inducing inflammation, also release IL-33, which in turn stimulates the mineralization of the bone matrix. IL-33 acts directly by stimulating mature osteoblasts (OB) and their bone marrow progenitors, the mesenchymal stem cells (MSC), inducing their differentiation and maturation. Despite the production of the receptor activator of nuclear factor kappa-Β ligand (RANKL) and the partial blockage of the production of osteoprotegerin (OPG) by activated osteoblasts, the prevalent effect of IL-33 on the skeleton is anti-osteoporotic. Arrows with “forbidden” signal mean that il-33 has anti-osteoporotic activities.

The IL-33/ST2 signaling pathway is implicated in bone turnover in physiological as well as pathological conditions. Figure 2 illustrates the role of IL-33 in bone remodeling. IL-33 inhibits RANKL-induced osteoclast formation through the regulation of anti-osteoclastic genes, such as interferon regulatory factor-8 (IRF-8), which can be blocked by an anti-ST2 monoclonal antibody [43]. IL-33 acts on all different types of bone cells [64]. Notwithstanding its involvement in various acute and chronic autoimmune and inflammatory diseases, notoriously accompanied by marked bone resorption such as osteonecrosis of the hip and rheumatoid arthritis, the IL-33/ST2 pathway has been suggested as an important protective signaling for the skeleton, decreasing bone resorption in the femur, through reduced osteoclastogenesis and stimulation of osteoblastic function [65]. In addition, IL-33 acts on the bone also indirectly, inducing the synthesis of antiosteoclastogenic cytokines capable of interfering with the production of M-CSF and RANKL [67]. IL-33 and ST2 are both expressed in the alveolar bone of the jaws and in the cells of the periodontal ligament, where they exert bimodal effects. However, in physiological conditions, IL-33 participates in the maintenance of the architectural structure of the alveolar bone in response to mechanical stimulations, by reducing the formation and activity of osteoclasts and inducing their apoptosis [68]. In estrogen α receptor knockout mice, an increase in IL-33 mRNA has been observed [30,64]. Although both IL33/ST2 and estrogens protect the skeleton from bone loss, mostly independently, at least in some skeletal segments (for example, the jaw but not the femur), ovariectomy induces IL-33/ST2-dependent bone resorption. IL-33 belongs to the IL-1 cytokine family, as well as IL-1 and IL-18, which can also affect bone remodeling, albeit with contrasting actions: IL-1 stimulates the production of RANKL from osteoblasts with consequent osteoclastogenesis, while IL-18, secreted by macrophages and osteoblasts, inhibits osteoclasts and stimulates osteoblast proliferation and bone formation [36,42,43,44]. Most of the in vitro studies and animal models on the effects of IL-33 on bone turnover show substantially opposite effects.

Conflicting observations about the role of IL-33 in bone biology have been derived from animal studies. Okragly et al. reported normal bone mineral density (BMD) and bone strength in IL-33 knockout (KO) mice but rapid bone loss in the cases of transgenic overexpression of full-length IL-33 in osteoblasts [71]. However, it is unclear whether this is a direct effect of IL-33 on the cells involved in bone resorption or whether it is an indirect effect due to other pro-inflammatory cytokines induced by IL-33. In contrast, ST2 deficient (ST2^−/−^) mice subjected to mechanical loading in alveolar bone have increased mechanical loading-induced bone resorption, number of osteoclasts, and expression of proresorptive markers, whereas exhibit reduced numbers of osteoblasts and apoptotic cells in periodontium and diminished expression of osteoblast signaling molecules compared to wild type mice [72], suggesting that IL-33/ST2 have anti-osteoclastogenic effects and reduce osteoclast formation and activity. IL-33^−/−^ and ST2^−/−^ mice have a reduction of innate lymphoid cells type 2 (ILC2s) in peripheral tissues, suggesting that IL-33 is an activator of ILC2s, which in turn exert immune-regulatory functions and protect mice from bone destruction [73,74]. IL-33- and ST2-knockout mice show homeostatic dysregulation of granulocyte responses in both the blood and bone marrow compartments, suggesting that IL-33 acts on many other cell types besides bone cells, all able to indirectly influence the bone. For example, IL-33 precedes IL-5 in regulating eosinophil commitment [75], contributing to the complex cytokine network driving both allergic and bone diseases [76]. Shulze et al. demonstrated that IL-33 directly stopped osteoclast formation from bone marrow precursor cells [70], and Zaiss et al. described a shift in the differentiation of myeloid bone marrow progenitors from osteoclasts to macrophages [77]. On the other hand, IL-33 also acts on osteoblasts, decreasing the expression of osteoprotegerin (OPG) and increasing the production of osteoclastogenic factors, with consequent induction of bone resorption under inflammatory conditions [78]. Some reports suggest that IL-33 stimulates matrix mineralization in vitro [79] but other authors have not confirmed this specific action [80].

IL-33 participates in Th2-mediated processes, thus stimulating osteoblast maturation and decreasing osteoclastogenesis [81]. Mun et al. [82] showed that lL-33 directly promotes osteoclast differentiation from human monocyte precursors, thus inducing bone resorption independently by the RANKL pathway. On the contrary, Kiyomiya et al. [83] demonstrated that IL-33 inhibits RANKL-dependent osteoclast formation. Similarly, Zhu et al. [84] showed that IL-33 exerts suppressive effects on osteoclast differentiation through the inactivation of nuclear factor of activated T-cell cytoplasmic 1 (NFATc1), the key regulator for RANKL-induced osteoclast formation, and Velickovic et al. [85] demonstrated that mice deficient in the IL-33 receptor display increased osteoclast formation and low bone mass. IL-33 stimulates the synthesis of anti-osteoclastogenic cytokines, such as IL-10 and IL-4, thus shifting the differentiation of osteoclast precursors towards macrophages and dendritic cells [86]. The possible osteoclastogenic action of IL-33 demonstrated in some in vitro studies [87] is quite weak in vivo compared to RANKL and substantially dependent on several variables, including the specific osteoclast progenitor subpopulation on which it acts. In fact, in the pool of myeloid progenitors, cell subsets with different responsiveness to IL-33 stimulation have been identified, partially explaining the apparently contrasting results in the literature [88]. Therefore, due to the pleiotropic action of IL-33 and the controversial results found in the literature, the overall effect of this cytokine on bone homeostasis is difficult to define.

Release of alarmins or DAMPs (danger-associated molecular patterns), including IL-33, from damaged cells is a relevant mechanism by which immune cells can be alerted of tissue damage, and alarmins play a key role in the development of acute or chronic inflammatory diseases, cancer and osteoporosis [89,90]. The cell deaths of chondrocytes, osteoblasts, and osteocytes play important roles in skeletal development, repair, as well as in the pathogenesis of osteoporosis. Enhanced osteoclastogenesis induced by osteocyte death and autophagy through the release of alarmins has been described [91]. Osteocyte cell death and autophagy released DAMPs by binding to their receptors on macrophages, dendritic cells, monocytes, and neutrophils, promoting the production of proinflammatory cytokines including TNF-α, IL-6, and IL-1, which induce the expression of RANKL in osteoblasts. As a consequence, osteoclast precursors differentiate into mature osteoclasts through RANKL/RANK signaling, therefore leading to bone resorption but also to replacement of damaged bone [57,92]. Therefore, the dual-function alarmin IL-33 can either exert beneficial skeletal effects, leading to bone tissue repair, or provoke deleterious inflammation, leading to osteoporosis [68]. IL-33 acts at cellular, molecular, and transcriptional levels to mediate pluripotent functions in bone remodeling and has potential therapeutic value to mitigate osteoporosis [69,93,94,95,96,97].

IL-33 exerts protective effects on bone, also through Treg recruitment and inhibition of NF-kB mediated gene transcription. In the nucleus, IL-33 binds to the acid pocket of the H2A–H2B dimeric histones on the nucleosome surface that suppresses gene transcription, allowing IL-33 interaction with the transcription factor NF-kB. The consequently compromised binding of NF-kB to DNA leads to a reduced NF-κB dependent pro-inflammatory gene transcription [98]. Foxp3+ Treg cells, in addition to the role in immune homeostasis, also exert critical functions in metabolic and regenerative processes, including the differentiation of hemopoietic stem cells and the function of osteoclasts. Moreover, the impact of Foxp3+ Treg cells on hematopoiesis is mediated by their regulatory action on osteoclast development and function, with consequent influence on the size and composition of the bone marrow niche. Niche-associated Treg cell subsets specifically interact with bone cells [99]. The modulation of Foxp3+ Treg cell function could represent a promising approach to restore bone homeostasis not only in inflammatory bone loss associated with rheumatic diseases but also in non-autoimmune contexts of aberrant bone remodeling, such as dysmetabolic, neoplastic, and drug-induced osteoporosis. 

## 4. Clinical Significance of Interleukin-31 and Interleukin-33 in Osteoporosis

The effect of the immune system on bone remodeling is mediated by cytokines: some cytokines contribute to bone resorption, while other cytokines exert protective effects, and still others perform pleiotropic functions [100,101,102]. IL-31 and IL-33 play essential role in osteoporosis but their final effect depends on multiple factors, including their mutual interaction and ultimately the combined action of the other cytokines and suppressor and/or stimulatory factors involved in the various phases of the complex bone remodeling process which controls the health of our skeleton [35,36,39,93].

Aging and lack of sex steroids regulate IL-31 and IL-33, as well as their receptors, in the bone environment. Post-menopausal osteoporosis is a clear example of the mutual influences between the immune, bone, and endocrine systems. Menopausal estrogen decline increases T-cell and macrophage activation, resulting in the production of inflammatory cytokines that are responsible for the chronic stimulation of osteoclast formation and consequent bone loss [9]. Maintenance and amplification of inflammatory reactions leading to osteoclastogenesis and increased risk of fractures are also present during senescence and inflammation, which is the condition of chronic inflammation characterizing aging [103].

During senescence, under the influence of the lifelong exposure to chronic antigenic load and oxidative stress, besides the impaired regulation of immune functions by Tregs, there is an increased number of effector memory cells, which are mainly senescent and proinflammatory cells secreting large amounts of proinflammatory cytokines, including IL-31, involved in the regulation of bone turnover [9]. Osteoblast number and activity decrease with aging, contributing to the age-associated decline of bone mass [104]. Inflammaging induces osteoporosis by promoting bone destruction and inhibiting bone formation [105]. An increase of IL-31 has been linked to decreased BMD in postmenopausal women. Binding of IL-31 to its receptor results in the phosphorylation and activation of STATs, MAPK, and JNK signaling pathways [66]. IL-31 plays a pathogenic role in tissue inflammation and bone destruction also during aging. Low-grade inflammation, due to the effect of pro-inflammatory cytokines, impairs DNA repair and leads to cellular and immunological senescence as well as biological aging [106]. However, the final effect of each inflammatory cytokine on bone remodeling depends on the specific cell target and the cytokine milieu in the bone. For example, IL-33 shifts the balance from osteoclast to alternatively activated macrophages differentiation and protects from inflammatory bone loss [77]. 

Aging impacts both Treg and the IL-31/IL33 axis, which are also important regulators of macrophage phenotypes and functions. Age-associated defects on macrophage polarization toward a pro-inflammatory (M1) or an anti-inflammatory (M2) phenotype characterized by specific markers, including Found In Inflammatory Zone 1 (Fizz1), have been demonstrated [107]. Sex steroid hormones modulate alternative macrophage activation by selectively regulating the expression of different genes associated with alternative macrophage activation [108,109]. Macrophages that acquire an alternative activation phenotype linked to type 2 immunity upregulate the IL-33R and express the inflammation-associated Fizz1 protein, whose gene is up-regulated by IL-31 [29,110].

The family of innate lymphoid cells has important roles in the regulation of inflammatory diseases, including osteoporosis. In particular, ILC2s show an IL-33-dependent proliferation and produce IL-5 and IL-13 responding to IL-33 and IL-25 [111]. In addition, TGF-β upregulates the expression of gene encoding for the IL-33 receptor ST2 in ILC2 progenitors. TGF-β signaling, which is required for the generation and maintenance of ILC2 progenitors, is altered in aging [112].

IL-33 also plays an important role in the recruitment and function of Tregs in mice. Tregs play an important role in the repair and regeneration of bone. IL-33 participates in Treg recruitment into the site of injury. Tregs inhibit M1 macrophage-mediated inflammation. In the bone, Tregs are most likely recruited via CCL22, which act on inhibiting Th1, CD8+, and M1 macrophages to support osteoblast progenitor differentiation. Tregs may also directly promote osteoblast differentiation from progenitor cells. For instance, it has been demonstrated that Tregs facilitate MSC-based bone regeneration by inhibiting CD4+ conventional T-cells, which secrete IFN-γ and TNF-α. In vivo experiments have also shown that Tregs protect TNF-α-induced bone destruction and ovariectomy-induced bone loss [113]. IL-33 drives accumulation of Tregs in damaged tissues in young mice by acting on the ST2 receptor of Treg [114]. IL-33(+) cells are reduced in old mice and fail to accumulate in injured tissues to drive repair processes. Normal repair of skeletal muscle, as well as bone, requires the IL-33-induced local expansion of a special population of Treg cells that fail to accumulate in damaged tissues of old mice, known to undergo ineffectual repair. Aged mice with more severely impaired muscle and bone repair have less IL-33-dependent accumulation of Treg after acute injury compared to young mice [115]. Thus IL-33 regulates Treg cell homeostasis in young mice, and its administration to old mice ameliorates their deficits in Treg cell accumulation and skeletal muscle and bone regeneration [116].

In clinical conditions, IL-33 could be regarded as a dual-function alarmin. It drives atopic inflammation and contributes to the development of several autoimmune diseases, whereas it is protective against infections [96] and atherosclerosis [97]. Therefore, depending on the disease, it can either drive immune-mediated pathologies or promote the resolution of the underlying inflammation and could have potential therapeutic value to mitigate the disease process [69,92,93]. Given osteoporosis is characterized by a Th1 immune response, the IL-33/ST2 axis could exert protective effects by inducing a switch from Th1 to Th2 immune responses [64]. Interestingly, cigarette smoking, which is a known osteoporotic risk factor, causes a dramatic decrease in ST2/IL-1R3 expression on Th2 cells and ILC2, by reducing the release of osteoprotective Th2 cytokines, including IL-4, IL-5, and IL-13. Although IL-33 apparently worsens arthritis and IL-33 blocking attenuates the disease progression in autoimmune conditions, the results of studies on IL-33 deficient mice indicate a rather modulating or even protective effect against the development of bone disease. Therefore, IL-33 can exert variable effects depending on the specific inflammatory context. Even the extracellular release of IL-33 by damaged cells during inflammatory stress can be either deleterious, such as in the context of allergic inflammation, or protective and repairing as in the case of osteoporosis. Taken together, these findings ultimately suggest that IL-33 is a pleiotropic cytokine whose peculiar function in bone is a strong induction of anti-osteoclastogenic Th2 cytokines [80].

The functional relationship binding IL-33 and estrogens in bone remodeling is still poorly understood. In postmenopausal osteoporosis, the decrease in estrogen levels is the basis of the accelerated bone turnover and the trabecular and cortical bone loss leading to a decrease in the BMD [117]. Estrogens directly inhibit RANK-induced osteoclastic differentiation, upregulate the secretion of osteoprotegerin (OPG) by bone marrow stromal cells and osteoblasts, and upregulate the production of IL-10 and semaphorin 3A (Sema3A) [118]. Moreover, the lack of estrogens also causes bone loss through the increase in the production of TNF-α, IL-1, and IL-6, with consequent upregulation of osteoclastogenesis [119]. Under physiological conditions, both IL-33 and estrogen protect vertebrae, long bones, and maxilla from bone loss. On the other hand, the osteopenic phenotype with decreased BMD observed in IL-33 knockout (IL-33^−/−^) mice confirms the osteoprotective role of IL-33. Moreover, the depletion of IL-33 or its receptor causes bone loss in the femur, vertebra, and maxillary bone in mice, suggesting that IL-33 controls both cortical and cancellous bone [64]. IL-10, upregulated by both IL-33 and estrogen, has a potent inhibitory effect on osteoclastogenesis. Unlike estrogens, IL-33 has little effect on TNF-α induced bone remodeling [58]. IL-33, together with the anti-inflammatory cytokine IL-10, is also related to the ferritin status in post-menopausal women, suggesting a role of these anti-inflammatory and anti-osteoclastogenic cytokines in the regulation of iron metabolism too [66]. Findings from IL-33^−/−^ and ST2^−/−^ mouse models of postmenopausal osteoporosis revealed that IL-33 and its receptor protects the maxilla and femur from bone loss during physiological bone remodeling. However, IL-33 expressed in the maxillary bone is associated with an exacerbated bone loss in a RANKL-dependent manner in the context of bacterial infection, such as in periodontitis, and ovariectomy-induced bone resorption is dependent on IL-33/ST2 signaling in the maxillae, but not in the femur or vertebrae, in a site-specific manner. This result might be linked to differential production of IL-33 in these bone sites induced by ovariectomy [64,70]. In a recent study investigating serum IL-33 levels in post-menopausal women, we showed a decrease of this cytokine in osteoporotic patients. We also demonstrated a negative correlation between IL-33 and the resorption marker CTX (C-terminal telopeptide of collagen type I), suggesting an inhibitory effect of IL-33 on osteoclastogenesis in vivo. The finding of low levels of IL-33 in postmenopausal osteoporosis is consistent with previous observations suggesting an inhibitory action of IL-33 on osteoclast differentiation [64]. The final effect of IL-33 on bone remodeling is conditioned by the clinical context: it varies according to the severity of the disease and changes in relation to hormonal influences. For example, IL-33 and its receptors are implicated in the control of bone turnover by parathyroid hormone (PTH), an important osteoanabolic factor when administered pharmacologically [120]. Saleh et al. [79] demonstrated that PTH and M oncostatin increase IL-33 mRNA levels in osteoblasts in culture. This result is in agreement with our observation of a positive correlation between serum PTH and IL-33 levels in postmenopausal osteoporosis. PTH stimulates the production of IL-33 which in turn participates in the osteoanabolic effects of this hormone. Furthermore, our observation of a positive correlation between IL-33 and the anabolic bone marker P1NP (N-terminal propeptide of type 1 collagen) also appears to support this hypothesis. Furthermore, although serum IL-33 levels are lower in menopausal women with osteoporosis than those with normal BMD, the likely protective effect of IL-33 on the bone tends to disappear as the disease progresses, probably due to the interference of other osteoclastogenic cytokines.

Vitamin D, together with PTH, is indispensable for the physiological bone turnover, and also performs important immunoregulatory functions. Vitamin D deficiency, very common in the elderly as well as in menopausal women, represents one of the main osteoporotic risk factors, and supplementation with vitamin D is recommended to prevent osteoporosis and to support anti-osteoporotic therapies. A deficiency of vitamin D is also the background of many inflammatory and autoimmune diseases, causing osteoporosis. It has been recently demonstrated that IL-33 and IL-31, together with IL-25, participate in the modulation of inflammatory processes involving vitamin D deficiency [121,122].

Th2 cytokines do not always positively affect the bone [123], but they may even contribute to the development of osteoporosis, as we demonstrated in a recent study, in which the Th2 cytokine IL-31 was significantly involved in osteoporosis development [35]. In particular, we have shown an increase in serum IL-31 levels in postmenopausal osteoporosis, although not correlated with the severity of the disease, as assessed by BMD values and presence of fragility fractures. In addition, higher levels of IL-31 were found in patients with more advanced age, suggesting an involvement of this cytokine in the processes driving immunosenescence and age-related diseases, particularly senile osteoporosis [38]. In fact, elderly subjects with osteoporosis have higher IL-31 serum levels than healthy young and old control subjects [35]. The increased IL-31 production by senescent inflammatory cells contributes to senile osteoporosis development. Increased IL-31 production might enhance bone resorption by enhancing the production of osteoclastogenic cytokines. IL-31, although belonging to the Th2 profile, induces the secretion of various inflammatory cytokines and matrix metalloproteinases, with consequent osteoclast production and bone resorption, suggesting the possibility of future anti-osteoporotic therapies against it. Overall, our studies have shown that IL-33 is decreased in osteoporosis, whereas IL-31 is increased. In general, it can therefore be suggested that IL-31 is osteoclastogenic, while IL-33 is protective, in the context of osteoporosis. 

Other authors have shown an involvement of the IL-33/ST2 axis in both the generation of Th17 cells and the production of IL-31 [27,124]. IL-31 and IL-33 are therefore closely linked: IL-31 dampens excessive Th2-type responses with detrimental effects on bone remodeling. Recent evidence suggests that the IL-33/IL-31 axis constitutes a link between accelerated atherosclerosis and osteoporosis in psoriatic arthritis (PsA) [30]. All these data also suggest that activation of the IL-33/ST2 axis may be considered as a biomarker of Th2/IL-31 and Th17 immune responses [36,125,126]. Inflammatory cytokines also play central roles in the development of neoplastic osteolysis. IL-33, produced from necrotic or inflamed tissues, and its ST2 receptor, are increased in patients with cancer. IL-31 has also been found to be increased in some cancers, such as breast cancer, whose main metastatic location is the skeleton [127].

The increase in IL-31 is associated with the reduction of BMD in postmenopausal women [35], while IL-33 appears to have a protective effect on bone loss [36]. However, the inflammatory process that accompanies inflammation and/or menopause is multifactorial, depending not only on immunosenescence and estrogen decline, but also on other factors such as dysmetabolisms, co-morbidities, drug assumption, intestinal microbiota status, and nutritional factors, and each of them somehow impacts on bone remodeling. There is a complex interaction between these two cytokines: while IL-4 induces the expression of IL-31 genes, IL-33 facilitates IL-31 release from Th2 lymphocytes [38]. The induction of IL-31 is mediated by IL-4/STAT6 and IL-33/NF-kB transduction signals, while it is downregulated by suppressor of cytokine signaling (SOCS), a family of genes involved in inhibiting the JAK–STAT signaling pathway [39]. Therefore, researchers have recently shifted the attention from the single molecule to the hypothesis of a structured functional axis [40,43]. The deepening of the knowledge of the roles of IL-31 and IL-33 in the immunoskeletal interface can contribute to better understanding of those mechanisms underlying the functioning of the immune system that trigger osteoclastogenic inflammatory reactions, crucial in osteoporosis.

## 5. Future Perspectives and Concluding Remarks

The close relationship that is emerging between IL-31 and IL-33 in the context of osteoimmunology and their mutual balance seem to be central to bone remodeling. Table 1 summarizes the main IL-31 and IL-33 functions in the context of osteoimmunology. IL-31 regulates a wide range of biological functions, induces proinflammatory cytokines, and controls cell proliferation and tissue remodelling. IL-33 is an alarmin released from cells of different tissues and organs after a damage signal. There is a close association between these two interleukins both at serum and tissue level and a functional correlation axis between them is emerging. Signals from different cell types modulate the activity of the IL-33/IL-31 axis [88]. The IL-33/ST2 axis, involved in several pathologies, influences the generation of Th17 cells that produce IL-31. Activation of the IL-33/ST2 axis could be therefore considered a good biomarker of the Th2/IL-31 and Th17 immune responses. The receptors of both cytokines, IL-31RA and ST2, are simultaneously expressed on fibroblasts, suggesting a synergistic stimulating action on these cells [29,39]. The final effects of the IL-33/IL-31 axis on bone is heavily determined by the complexity underlying their reciprocal influences. The impact of other inflammatory cytokines and costimulatory signals, as well as the peculiar clinical context of the osteoporotic condition, influences the final effect of IL-31 and IL-33 on bone turnover. The specific role of the IL-33/IL-31 axis in the context of osteoporosis is, therefore, an intriguing matter [128]. In the majority of inflammatory diseases, the IL-31 and IL-33 pathways are linked to each other, with a significant positive correlation between their expression and disease severity [39]. The increased expression of one of them could induce the other one, leading to amplifying inflammation and disease progression. However, in osteoporosis, a different and more complex situation seems to occur: while IL-31 levels positively correlate with disease severity, IL-33 is inversely correlated. In the future, the goal will be to influence their reciprocal balance, so as to modulate both inflammatory responses and bone turnover. Many clinical and experimental observations suggest an important role of IL-31 in osteoporosis onset and progression. In vitro studies show the influence of IL-31 on the differentiation of Th1 and Th17 lymphocytes. IL-31 regulates immune and inflammatory responses indirectly through a remodulation of antigen-presenting cells and directly through the negative regulatory signal IL-31/IL-31R on T cells, thus specifically limiting Th2 inflammatory processes, which are substantially protective in osteoporosis [80]. On the contrary, IL-33 inhibits RANKL-dependent osteoclastogenesis, thus counteracting the loss of bone mass that accompanies inflammatory conditions [129,130].

Given their importance in the pathogenesis of osteoporosis, both IL-31 and IL-33 could be used as biomarkers in the diagnosis and follow-up of osteoporotic patients. Changing the IL-31/IL-33 balance could interfere with the initial immunological responses underlying the onset and progression of osteoporosis. Targeted biological therapies [50,131,132], such as monoclonal antibodies and fusion proteins against IL-31 and IL-33 cytokines or their receptors, are already being tested in some allergic diseases. IL-31, responsible for the itching in atopic dermatitis, has been the first therapeutic target. In particular, the administration of nemolizumab (anti-IL-31RA antibody) seems effective and safe in the treatment of itching. Bispecific antibodies to both IL-31 and IL-33 receptors are in development. On the other hand, IL-33 acts as an alarmin: necrotic cells release the integral bioactive form of IL-33, which functions as an endogenous warning signal. The increased expression of IL-33 after cell death causes the induction of other cytokines, including IL-31 [133,134,135]. In particular, IL-33 promotes IL-4 dependent release of IL-31 by CD4+ Th cells. As IL-33 is considered an osteoprotective cytokine, it could become a useful pharmacological tool in the prevention and treatment of osteoporosis. However, as a therapeutic target, it could be a double-edged sword because of its multivalent functions.

## Figures and Tables

**Table 1:** Summary of IL-31 and IL-33 functions in osteoimmunology.

IL	Cytokine Family	Expression	Homeostatic Function	Receptors	Effects on Bone
IL-31	IL-6 family cytokine	Activated monocytes, myeloid progenitors, macrophages, eosinophils dendritic and CD45RO+ Th2 cells	Multiple regulatory functions on cell cycle and tissue remodeling, tissue damage and repair processes; Th2 response induction; EGF and VEGF upregulation	IL-31RA/OSMR	OPC proliferation; monocyte/macrophage activation; increased proinflammatory cytokine gene transcription, production of osteoclastogenic cytokines, matrix metalloproteinases and chemokines; Th1/Th17 lymphocyte differentiation; APC function regulation; tumorigenesis and bone metastases [29,38,102]
IL-33	IL-1 family cytokine; alarmin	Multiple organs and cell types (mainly bone marrow stromal cells, fibroblasts, epithelial and endothelial cells) following pro-inflammatory stimulation	Alerting for tissue damage; gene transcription control; immune response amplification and tissue repair during cell injury; Th2 cell maturation and chemoattraction; Th2-associated cytokine induction; eosinophil activation and mast cell degranulation; transcription of genes involved in inflammatory responses, including IL-31; Foxp3+ Treg cell induction	IL1RL1/ST2	Repression of NF-kB transcription; NFATc1 inactivation; inhibition of RANK/RANKL signaling; bone resorption decrease; OC apoptosis induction; OB function stimulation; anti-osteoclastogenic cytokine induction; maintenance of architectural bone structure in response to mechanical stimulations; OC to macrophage switch in myeloid bone marrow progenitors [29,34,108,109]

Interleukin (IL); T helper (Th); osteoclast precursor cell (OPC); osteoclast (OC); osteoblast (OB); antigen presenting cell (APC); epidermal growth factor (EGF); vascular endothelial growth factor (VEGF); T regulatory cell (Treg); nuclear factor-kB (NF-kB); nuclear factor of activated T-cells, cytoplasmic 1 (NFATc1).
